# The surgical management of fracture-related infection. Surgical strategy selection and the need for early surgical intervention

**DOI:** 10.1016/j.jor.2023.11.033

**Published:** 2023-11-29

**Authors:** Leonard C. Marais, Charalampos G. Zalavras, Fintan T. Moriarty, Richard Kühl, Willem-Jan Metsemakers, Mario Morgenstern

**Affiliations:** a Department of Orthopaedics, School of Clinical Medicine, University of KwaZulu-Natal, Durban, South Africa; b Department of Orthopaedic Surgery, Keck School of Medicine, University of Southern California, Los Angeles, USA; c AO Research Institute Davos, Davos, Switzerland; d Center for Musculoskeletal Infections, Department of Orthopaedics and Trauma Surgery, University Hospital Basel, Basel, Switzerland; e Department of Infectious Diseases and Hospital Hygiene, University Hospital Basel, Switzerland; f Department of Trauma Surgery, University Hospitals Leuven, Leuven, Belgium; g Department of Development and Regeneration, KU Leuven, Leuven, Belgium

**Keywords:** Fracture-related infection, Postoperative infection, Orthopaedic trauma, Osteomyelitis, Management, Surgery

## Abstract

The aim of this narrative review is to describe the various surgical management strategies employed in fracture-related infection (FRI), to explore how they are selected and discuss the rationale for early surgical intervention. Surgical treatment options in patients with FRI include debridement, antibiotics and implant retention (DAIR), revision (exchange) or removal. In selecting a treatment strategy, a variety of factors need to be considered, including the condition of the bone, soft tissues, host and causative microorganism. Irrespective of the selected treatment strategy, prompt surgical intervention should be considered in order to confirm the diagnosis of an FRI, to identify the causative organism, remove necrotic or non-viable tissue that can serve as a nidus for ongoing infection, ensure a healthy soft tissue envelope and to prevent the vicious cycle of infection associated with skeletal and/or implant instability. Ultimately, the objective is to prevent the establishment of a persistent infection. Urgent surgery may be indicated in case of active, progressive disease with systemic deterioration, local progression of infection, deterioration of soft tissues, or progressive fracture instability. In case of static disease, the patient should be monitored closely and surgery can be performed on an elective basis, allowing adequate time for optimisation of the host through risk factor modification, optimisation of the soft tissues and careful planning of the surgery.

## 1 Introduction

Fracture-related infection (FRI) is one of the most common major complications in orthopaedic trauma surgery and also one of the most challenging to treat. It has been identified as a global health concern and harbours substantial implications for both the patient and the healthcare system.^1^^,^^2^ Up to 30 % of high grade open fractures may develop FRI and this has been shown to result in a significant decrease in patients’ health-related quality of life.^3^^,^^4^ The impact of an FRI is not only felt in the short term; four years after successful treatment patients still suffer a significant reduction in their quality of life.^5^ Furthermore, FRIs may result in a six-fold increase in healthcare costs.^6^^,^^7^ Treatment can be protracted, particularly for post-infective bone defects, resulting in patients suffering loss of income, psychological stress, physical discomfort and ultimately functional impairment.^8^ Despite this significant burden there is still limited high-level evidence available to guide the treatment of FRI.^9^^,^^10^

Recently a systematic literature review examining the outcomes of FRI treatment reported overall success, with bony union and eradication of infection, in 85 % with 9 % recurrence.^9^ The authors emphasized the heterogeneity of management protocols in the literature and the need for establishing standardized outcome measures. Over the past few years significant strides have been made in standardisation of the definition and diagnosis of FRI. An international FRI consensus group was established in 2016 under the auspices of the Arbeitsgemeinschaft fur Osteosynthesefragen (AO) Foundation, in collaboration with other international organisations such as the Orthopaedic Trauma Association (OTA), the European Bone and Joint Infection Society (EBJIS) and the PRO-IMPLANT Foundation.^11^ This group established the standardized definition and evidence-based diagnostic criteria, that were evidently needed by both clinicians and researchers in the field as suggested by the fact that the term and diagnostic criteria has been widely adapted in the literature and in practice.^12^

Despite this progress, gains in the outcome of treatment have been described as remaining moderate.^13^ The general treatment principles are well established, including a multidisciplinary approach, host optimisation, appropriate sampling, judicious debridement, skeletal and soft tissue reconstruction and the use of local and systemic antibiotics.^14^ However, how exactly these strategies should be implemented on an individual patient basis is less clear and there are variable ways in which these principles may be applied in practice. Every FRI case is different depending on the patient's physiological status, the anatomic location and healing status of the fracture, the presence of bone defects, the fracture fixation, the condition of the soft tissues, and the causative organism. Selecting the appropriate treatment strategy and choosing the appropriate time to intervene, particularly in terms of the surgical management, can be difficult and there is little clinical evidence to aid in the decision making process.

The aim of this narrative review is to describe the surgical management strategies employed in FRI, to explore how they are selected and discuss the rationale for early surgical intervention.

## 2 Rationale for early surgical intervention

It is prudent to consider surgical intervention for all patients with a suspected FRI irrespective of the time to onset of symptoms or the duration of the infection. The aims of surgery are to: (1) confirm the diagnosis of FRI, (2) identify the offending organism through appropriate sampling, (3) remove non-viable tissue that can serve as a nidus for ongoing infection and decrease the bacterial load, thus giving the antibiotics the best chance of success, (4) ensure a healthy soft tissue envelope that can serve as an effective barrier to further contamination and create a biological environment conducive to fracture healing and infection control, and (5) to prevent the vicious cycle of infection associated with implant and/or skeletal instability. Ultimately the objective of the early surgical intervention is to prevent the establishment of a persistent infection.

The first motivation for early surgical intervention is to confirm the diagnosis of a FRI. The Centres for Disease Control and Infection (CDC) and National Healthcare Safety Network (NHSN) in the US classifies of surgical site infections as either superficial incisional, deep incisional or organ/space infection.^15^ This classification was, however, designed for surveillance purposes and not clinical decision making. A secondary analysis of the FLOW (Fluid Lavage of Open Wounds) study cohort suggested that nonoperative management with antibiotics may resolve superficial surgical site infection involving only skin or subcutaneous tissue (SSI).^16^ These findings suggest that differentiating between superficial and deep infection may possibly be useful in clinical practice. However, distinguishing superficial site infection from deep surgical site infection is often challenging and there is no evidence-based, non-invasive means of reliably discerning one from the other.^17^ And in certain anatomic locations, such as the distal fibula, it is likely that an infection involves the deeper structures and communicates with the implant. The price of misdiagnosing a deep infection as a superficial one may be high, as a delay in appropriate treatment may result in establishment of a persistent infection. Therefore, a pragmatic approach involving surgical exploration to confirm the diagnosis of FRI (i.e., a deep infection) appears to be justified in certain cases.

The diagnosis of FRI should be made on the basis of confirmatory criteria, as defined by the FRI consensus group.^12^ Clinical confirmatory criteria are the presence of a fistula, sinus, or wound breakdown (with communication to the bone or implant) and/or purulent drainage from the wound or presence of pus during surgery.^18^ In the absence of these clinical features, the diagnosis can only be confirmed with culture of phenotypically indistinguishable microorganisms from at least two separate deep tissue/implant specimens, and/or the identification of microorganisms through specific staining techniques or the presence of at least 5 neutrophils per high-power field (HPF) on histopathological examination.^19^ The implication is that, in the absence of clinical confirmatory criteria, the diagnosis can only be made by appropriate surgical sampling.

The second reason for prompt surgical intervention is the need to identify the causative organism. Superficial, skin, or sinus tract samples have little diagnostic value and should be avoided, as these will grow commensal microorganisms with no predictive value for the causative pathogen of FRI.^20^ Appropriate sampling involves five or more deep tissue or fluid samples collected from the implant–bone interface, and if available sonication of the implant in case the implant is removed.^21^ Accurate diagnosis of the causative bacteria and their antibiotic resistance profile can then appropriately inform the selection of antibiotic therapy.^22^

Early debridement aims to reduces the bacterial load and remove all non-viable tissue, reducing the risk of bacterial persistence. In early FRIs, Morgenstern et al. has shown that an increase in the duration of the infection is associated with an increased risk of failure of treatment when implants are retained.^23^ This is reminiscent of the situation in severe open fractures, where the current evidence suggests that a delay to debridement is associated with an increased risk of the development of an FRI.^24^

Establishing a healthy, well-perfused soft tissue envelope provides the ideal environment for fracture healing, an effective local immune response to infection and optimal delivery of antibiotics, oxygen and nutrients (Table 1). In addition, promptly establishing a robust physical and immunological barrier may prevent further contamination and exposure to nosocomial pathogens. While there is limited evidence to provide clear timelines, the available data suggests that undue delay in soft tissue coverage should be avoided. A parallel can again be drawn with open fractures, where evidence suggests that a delay in soft tissue coverage of more than 7 days results in an increased risk of infection.^25^^,^^26^

**Table 1:** Aims of treatment of FRI.^18^.

1.	Fracture consolidation
2.	Eradication of infection or in certain cases suppression of infection until fracture consolidation
3.	Healing of the soft tissue envelope
4.	Prevention of chronic osteomyelitis
5.	Restoration of functionality

Stability of the fracture is a critical factor in the management of FRI, not only in order to ensure fracture union but also to avoid bacterial proliferation and persistence.^27^ Instability leads to ongoing soft tissue trauma and disrupts neovascularization; resulting in inflammation, impaired local immune response and reduced penetration of antibiotic agents. On a biomechanical level it leads to osteolysis, bone resorption and implant loosening. This leads to a vicious cycle with increasing instability and bacterial proliferation. Thus, any instability should be addressed as soon as possible to interrupt this cycle.

The final motivation for prompt surgical intervention in FRI is to prevent the establishment of a persistent infection that will be more difficult to eradicate. While significant emphasis has in the past been placed on the role of biofilms in the pathogenesis of FRI, it is not the only mechanism by which infecting organisms can evade immune defence and diminish antibiotic efficacy.^28^ Osteoblasts, osteoclasts and professional phagocytes, like macrophages, may serve as reservoirs for intracellular bacteria.^28^ Invasion of the osteocyte lacuno-canalicular network may be another key factor in bacterial persistence through evasion of antibiotics and the immune response.^29^ These “hubs” may serve as the nidus of continued bacterial invasion. Finally, abscesses, for example in the medullary cavity, may also serve as bacterial reservoirs offering protection against antibiotics. This all occurs in the background of a complex infectious microenvironment, reminiscent of the microenvironment seen in tumours.^30^

Our knowledge of biofilms is mostly based on the results of in vitro experimentation. In vivo biofilms however differ significantly from what is predicted by current in vitro models. The main reason is the absence of immunological defence mechanisms in the in vitro models. In vitro studies suggest that staphylococcal biofilm formation can occur within 24–48 h of bacterial contamination and these biofilms are already antibiotic tolerant.^31^ *Staphylococcus aureus* biofilm formation has been illustrated as early as one week following infection in an animal model.^32^ Li et al. demonstrated that although in vivo biofilm formation is initiated within 48 h after inoculation, the resulting acquired immune response starts limiting bacterial growth to a biofilm growth pattern from about day 10.^33^ The term “race for the surface”, which refers to the competition between bacterial colonisation and host defence mechanisms, has become synonymous with implant related infections.^34^ Yu et al. have shown that Methicillin-resistant *S. aureus* can infiltrate osteoblasts within an hour of exposure.^35^ Furthermore, Shiels et al. showed that the infection rate and bioburden decreases as the time between implantation and bacterial contamination increases.^36^ It appears that, after approximately one week, the host-cell integration may often be sufficiently competent to prevent infection. Taken together, these observations again places importance on early intervention in order to prevent bacterial persistence.

**Fig. 1:**
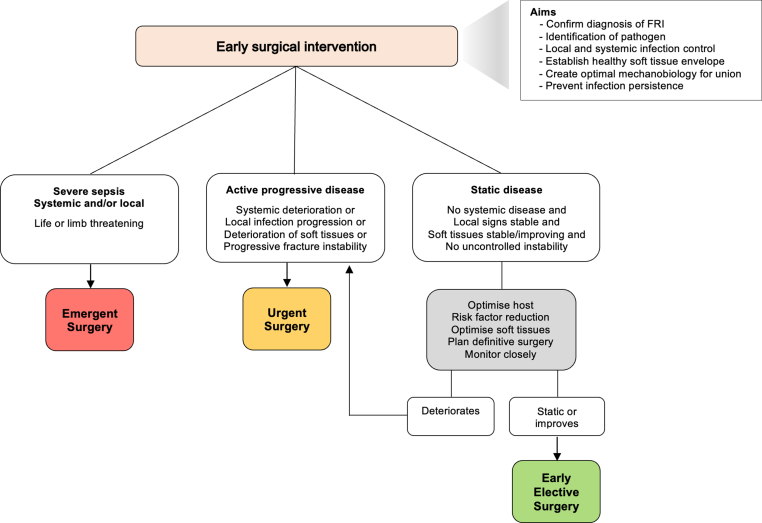
Indications for, and aims of, early surgical intervention in fracture-related infection. The timing of surgery is based on specific indications, and can be either emergent, urgent or early elective. Early elective surgery allows opportunity for host and soft tissue optimisation, as well as surgery planning, but without further unwarranted delay.

While the rationale behind early surgical intervention appears evident, the indications for emergent/urgent surgery are less distinct. Clear-cut indications include sepsis or severe systemic infection, severe local infection, deteriorating soft tissues and/or a highly unstable fracture [Fig. 1]. In case of static disease the patient should be monitored closely and early surgery can be performed on an elective basis, allowing adequate time for host optimisation through risk factor modification, optimisation of the soft tissues and careful planning of the surgery.

## 3 Surgical treatment strategies

Surgery remains one of the cornerstones of the treatment of FRI.^22^ All surgical approaches have one important element in common, a judicious well-planned debridement with removal of all dead tissues and acquisition of deep tissue biopsies for microbiology and histopathology.^29^ Local and systemic antibiotics are valuable adjuncts, but alone are unlikely to ensure eradication of infection.^37^ Following the debridement and sampling, the fracture fixation needs to be addressed. This can either be removed, retained or revised.^38^ Repair or reconstruction of the soft tissue envelope is essential, irrespective of what approach is used in terms of the fracture fixation and skeletal reconstruction i.e. whether the initial implant is retained, exchanged or removed. A thorough debridement entails excising all necrotic or ischaemic tissue, including scarred soft tissue.^39^ The soft tissue envelope then needs to be reconstructed with well-vascularized tissue. This can be done at the same time as the debridement (one stage procedure) or as a subsequent surgical procedure (two stage soft tissue reconstruction).^40^^,^^41^

Selecting the appropriate surgical strategy depends on numerous factors related to the patient, pathogen, bone and soft tissue (Table 1).^42^ In selected cases, where fractures are judged to be in the optimal mechanical and biological environment to unite, the infection may be suppressed and implants retained until fracture union. In these scenarios, the subsequent surgery is much less complex when the fracture is united. If there is an established non-union present or if fracture union is deemed unlikely to occur, exchange/revision of the fixation is indicated. Exchange of the fixation is typically performed as either a single or a two-stage procedure. This approach can be likened to the one- or two-stage revision in periprosthetic joint infection (PJI).^43^

### 3.1 Implant removal

If the fracture is united, treatment is typically less complex because of the absence of instability; thus there is no need to perform internal or external fixation. This is one of the main reasons why achieving fracture union remains the main priority when dealing with FRI in the setting of an ununited fracture (Table 1).^9^ In the case of a healed fracture, either a curative approach or palliative approach can be followed. In the vast majority of cases a curative strategy is employed, which involves debridement and implant removal. Very rarely, when the patient is deemed unfit for surgery, a palliative treatment strategy may be indicated. In this scenario, suppressive antibiotics are typically administered for a limited period of time while the patient is optimised for surgery.^44^^,^^45^ Rarely, long-term suppression or even amputation may be indicated when patient cannot be optimised for surgery. In the absence of union, the choice is between retention or revision of the fixation.

### 3.2 Implant retention

Retention of the original implants is often tempting, as it would negate having to redo the fixation, which could involve unintentional loss of reduction, additional soft tissue stripping or even further bone loss. However, the reported success rates of debridement, antimicrobial therapy, and implant retention (DAIR) are highly variable and dependant on a number of factors. An adequate debridement resulting in reduced bacterial burden and a vital well-perfused soft tissue envelope, are prerequisites.^14^ The first priority in the management of FRI is fracture union (Table 1), therefore the original implants should only be retained if the fracture is stable and there is a high likelihood of fracture union under the prevailing mechanical and biological environment Once this decision is made, the other factors associated with increased risk of treatment failure with implant retention need to be considered.

With an increase in the duration of the infection and the time from the index surgery, there is a corresponding rise in the failure rate due to recurrence of infection.^23^ Other factors associated with an increased risk of failure include: local or systemic compromising host factors (such as smoking, diabetes, or peripheral vascular disease), the location and nature of the injury (with lower extremity and open fractures being at higher risk), the type of implant present (intramedullary nails being associated with a higher risk), multiple debridements, failure to obtain primary soft tissue closure, the use negative pressure wound therapy following the debridement and difficult-to-treat pathogens (such as rifampin-resistant staphylococci or bacteria resistant to biofilm active antibiotics).^46^, ^47^, ^48^, ^49^, ^50^ No single factor should be seen as an absolute contra-indication for implant retention, but each case should rather be assessed with cognisance of all these risk factors.

### 3.3 Implant revision

If implant retention strategy is not deemed advisable, further decisions need to be made with regards to: 1) The need to reconstruct a bone defect, 2) The type of fixation that will be used following removal of the original implants, and 3) The timing of the exchange of fixation, i.e. whether it will be done in a single sitting or in stages.

The first question to ask when implant exchange is being considered is: Is there a critical bone defect? A critical bone defect can be defined as any defect that will not heal without further surgical intervention.^51^ The treatment of post-infective bone defects is complex; there are numerous treatment options and many factors that influence the decision making. There is currently limited robust evidence to guide treatment strategy selection in the management of bone defects and in many cases is based on the surgeons experience and preference.^52^ The choice of reconstruction option is dependent on the site, size and shape of the defect, the quality of the soft tissues and bone, and the presence of deformity, joint contractures or leg length discrepancy.

Treating large (>5 cm) segmental bone defects in the lower limbs is particularly challenging. While there was initially significant enthusiasm about the induced membrane technique, as popularized by Masquelet, this has waned with studies showing complication rates of up to 50 %,^53^, ^54^, ^55^ Concern has been raised about the use of this technique in segmental tibial bone loss, in particular, having been shown to have a low success rate.^56^ Recently, Feltri et al. showed in their meta-analysis on diaphyseal bone defects (not exclusively resulting from infection) that bone transport yielded the highest rate of primary union (91 %), as well as the lowest reintervention rate, when comparing it with bone graft procedures.^57^ However, the limitation of this study was that a heterogeneous group of cases were pooled together involving a variety of diagnoses and defect sizes. Another meta-analysis has shown that the use of distraction osteogenesis is associated with a high rate of resolution of infection for the treatment of long bone defects in the lower limbs.^58^ However, bone transport is a complex, lengthy procedure with a high demand being placed on both the surgeon and the patient.^59^ Vascularized free fibula autograft is another option to consider for large segmental defects, particularly in the upper limb.^60^^,^^61^ Potential advantages include a shorter time to union and lower number of surgeries, however fracture and fracture remains a concern.^62^

The next question to answer is: What type of fixation that would most reliably achieve union at the fracture site? The choice of fixation is also influenced by a variety of other factors, including the anatomic location of the fracture, the fracture configuration, the bone quality, the presence of a bone defect and the soft tissue envelope. If there is a critical bone defect the fixation would typically depend on the techniques selected to treat the bone defect. Historically, the prevailing thinking was that infection should be considered a contra-indication for internal fixation. However, Rittman and Perren illustrated in an experimental model, that bone healing can occur using internal fixation in the presence of infection; as long as the implant was stable.^63^ Others have emulated this work, showing that stable internal fracture fixation prevents the development of symptomatic infection despite bacterial contamination.^64^, ^65^, ^66^ This prompted Perren to state that “… the advantages of direct healing in the presence of infection, particularly the avoidance of sequestration, deserve close attention".^67^ While infection may not be an absolute contra-indication for internal fixation, there are limited data to support any theoretical advantage it may offer. Good results have been reported with the use of external fixation in the setting of post-infective bone reconstruction. Yin et al. showed in their meta-analysis that the use of Ilizarov methods resulted in poor bone or functional outcomes in less than 10 % of infected tibial or femoral non-unions.^68^

Fracture union remains the priority in the treatment of FRI. Therefore the fixation method that would most reliably yield fracture union should be selected. For this reason internal fixation may be an option when performing implant exchange in certain cases of FRI involving the upper limb. There is however a lack of comparative data with regards to the optimal method of fixation. McNally et al. have looked at the factors associated with failure of treatment in FRI and noted comparable failure rates with exchange to new internal fixation and conversion from internal to external fixation (12.5 % vs 10.3 %).^69^ The authors have previously proposed that internal fixation can be considered in selected patients, who are Cierny & Mader type A hosts, with less than 2 cm bone defects, good bone stock and soft tissue quality, and no multi-resistant organisms. They noted that, in their experience, internal fixation was used in less than 5 % of infected non-unions.^70^ With the increased use of local antibiotics there has, however, been an increase in the use of internal fixation in infection, for example with the use of antibiotic coated intra-medullary nails.^71^

The final question when embarking on an implant exchange treatment strategy is whether a single stage procedure is advisable. In a case with acute severe systemic or local sepsis (such as significant pus accumulation or significant cellulitis) a staged approach may be more appropriate and less risky. In its absence, the decision is not as simple. There are several other factors that may also influence the decision to embark on a staged approach, like concerns about the viability of the remaining bone, poor quality soft tissues, significant host impairment due to comorbidities and a multi-resistant difficult-to-treat pathogen.

A systematic review of a heterogenous group of studies involving infected non-union of long bones, published in 2007, noted comparable results with single or two stage procedures.^72^ One-stage strategies yielded union rates of 70–100 % and recurrence infection in 0–55 % of cases. On the other hand, two-stage strategies were associated with union rates of 66–100 % and infection recurrence varied from 0 to 60 %. However, the extensive heterogeneity of studies reviewed does not allow any recommendations to be made. More recently, cases series utilizing either one- or two-stage procedures have shown good results. McNally reported eradication of infection in 96 % (76 of 79) of tibial nonunions treated with a single stage procedure and circular external fixation at a mean follow-up of 40 months.^73^ Union was ultimately achieved in 91 % of patients. On the other hand, Zhang et al. reported a union rate of 90 % at 2-year follow-up with a staged approach in infected nonunion of long bones. Union occurred in 91 % of cases with negative cultures at time of definitive fixation, compared to 63 % of cases who were culture positive.^74^

## 4 Conclusion

Surgical treatment options in patients with FRI include implant retention (DAIR), revision (exchange) or removal. Selecting the most appropriate surgical treatment strategy can be challenging. During the treatment strategy selection process a variety of factors need to be considered, including the condition of the bone, soft tissues, host and causative microorganism. The bone and/or the soft tissue reconstruction procedures may need to be performed in a staged manner. Irrespective of which treatment strategy is selected, early surgical intervention is needed in order to confirm the diagnosis of an FRI, to identify the causative organism, and to prevent bacterial persistence.
